# A *Wohlfahrtiimonas chitiniclastica* with a novel type of *bla*_VEB–1_-carrying plasmid isolated from a zebra in China

**DOI:** 10.3389/fmicb.2023.1276314

**Published:** 2023-11-02

**Authors:** Jiayao Guan, Wei Zhou, Jingyi Guo, Lin Zheng, Gejin Lu, Fuyou Hua, Mingwei Liu, Xue Ji, Yang Sun, Lingwei Zhu, Xuejun Guo

**Affiliations:** ^1^College of Veterinary Medicine, Jilin Agricultural University, Changchun, China; ^2^Key Laboratory of Jilin Province for Zoonosis Prevention and Control, Changchun Veterinary Research Institute, Chinese Academy of Agricultural Sciences, Changchun, China; ^3^Center for Animal Disease Control and Prevention of Ordos, Ordos, China; ^4^The Second Hospital of Jilin University, Jilin University, Changchun, China; ^5^Shenzhen Safari Park, Shenzhen, China

**Keywords:** *Wohlfahrtiimonas chitiniclastica*, *bla*_VEB–1_, plasmid, drug resistance, zoonotic bacteria, mobile genetic element

## Abstract

**Background:**

*Wohlfahrtiimonas chitiniclastica* is an emerging fly-borne zoonotic pathogen, which causes infections in immunocompromised patients and some animals. Herein, we reported a *W. chitiniclastica* BM-Y from a dead zebra in China.

**Methods:**

The complete genome sequencing of BM-Y showed that this isolate carried one chromosome and one novel type of *bla*_VEB–1_-carrying plasmid. Detailed genetic dissection was applied to this plasmid to display the genetic environment of *bla*_VEB–1_.

**Results:**

Three novel insertion sequence (IS) elements, namely IS*Woch1*, IS*Woch2*, and IS*Woch3*, were found in this plasmid. *aadB*, *aacA1*, and *gcuG* were located downstream of *bla*_VEB–1_, composing a gene cassette array *bla*_VEB–1_–*aadB*–*aacA1*–*gcuG* bracketed by an intact IS*Woch1* and a truncated one, which was named the *bla*_VEB–1_ region. The 5′-RACE experiments revealed that the transcription start site of the *bla*_VEB–1_ region was located in the intact IS*Woch1* and this IS provided a strong promoter for the *bla*_VEB–1_ region.

**Conclusion:**

The spread of the *bla*_VEB–1_-carrying plasmid might enhance the ability of *W. chitiniclastica* to survive under drug selection pressure and aggravate the difficulty in treating infections caused by *bla*_VEB–1_-carrying *W. chitiniclastica*. To the best of our knowledge, this is the first report of the genetic characterization of a novel *bla*_VEB–1_-carrying plasmid with new ISs from *W. chitiniclastica*.

## 1. Introduction

*Wohlfahrtiimonas chitiniclastica* is an emerging fly-borne pathogen that causes bacteremia (Rebaudet et al., 2009), sepsis (Almuzara et al., 2011), cellulitis (de Dios et al., 2015), osteomyelitis (Suryalatha et al., 2015), wound infections (Nogi et al., 2016), and soft tissue and bone infections (Kõljalg et al., 2015) in humans and bacteremia (Thaiwong et al., 2014), endocarditis (Josue et al., 2015), and hoof fetlow (Qi et al., 2016) in animals. It was first identified from both the homogenate of the third-stage larvae of the parasitic fly *Wohlfahrtia magnifica* and the foregut of a third-stage maggot of *W. magnifica* collected from an infested vulval wound of a Romney sheep in Hungary in 2008 (Tóth et al., 2008). Since then, *W. chitiniclastica* has been associated with several obligate parasitic flies, including *W. magnifica* (Tóth et al., 2008), *Chrysomya megacephala* (Cao et al., 2013), *Lucilia sericata* (Campisi et al., 2015), *Musca domestica* (Gupta et al., 2012), and *Lucilia illustris* (Iancu et al., 2020). These flies can transmit *W. chitiniclastica* to humans and animals, which causes local or systemic infections originating from wounds infested with fly larvae. Diverse risk factors have been identified among infected patients and animals, including homelessness, alcoholism, tobacco abuse, poor hygiene, chronic open wounds, proximity to livestock, increasing age, low socioeconomic status, immunocompromised patients, chronic cardiovascular/circulatory disease, and neurological disease (Schröttner et al., 2017). To date, *W. chitiniclastica* has been identified in 10 countries in Europe (Tóth et al., 2008; Rebaudet et al., 2009; Campisi et al., 2015; Kõljalg et al., 2015; Schröttner et al., 2017; Pablo-Marcos et al., 2019; Dovjak et al., 2021; Hladík et al., 2021; De Smet et al., 2022; Karaca et al., 2022), four in Asia (Suryalatha et al., 2015; Zhou et al., 2016; Suraiya et al., 2017; Katanami et al., 2018), two in South America (Almuzara et al., 2011; Matos et al., 2016), one in North America (Thaiwong et al., 2014), one in Oceania (Connelly et al., 2019), and one in Africa (Hoffman et al., 2016).

It is speculated that *W. chitiniclastica* is intrinsically resistant to fosfomycin due to the fosfomycin efflux proteins, the gene homolog encoding for transferases, and the gene homologous to *mdtG* that related to fosfomycin resistance (Kopf et al., 2021). In addition, research has revealed that *W. chitiniclastica* shows resistance to amikacin (Kopf et al., 2021), tetracycline (Snyder et al., 2020), and tobramycin (Kopf et al., 2021), and it is intermediate to ampicillin (Qi et al., 2016). However, *W. chitiniclastica* is susceptible to the majority of the known antibiotics including β-lactams, quinolones, and trimethoprim/sulfamethoxazole (Kopf et al., 2022). Therefore, these three categories of antimicrobials may be the best options for treating *W. chitiniclastica* infections (Schröttner et al., 2017).

*bla*_VEB–1_ was initially identified from a clinical *Escherichia coli* isolated from Vietnam, and it encodes Vietnamese extended-spectrum β-lactamase (VEB-1) that displays high-level resistance to amoxicillin, piperacillin, cefotaxime, ceftazidime, and aztreonam (Poirel et al., 1999). Since then, *bla*_VEB–1_ has been found in IncA/C (Papagiannitsis et al., 2012), IncF (Paul et al., 2017), IncK (Paul et al., 2017), and IncP plasmids (Siebor et al., 2021) and in plasmids with undetermined Inc type from various Gram-negative species, including *Enterobacteriaceae* (Poirel et al., 1999), *Morganellaceae* (Oikonomou et al., 2016), *Acinetobacter* (e.g., *Acinetobacter baumannii* with the accession number LC107425), *Aeromonas* (e.g., *Aeromonas caviae* with the accession number KU886278), *Shewanella* (e.g., *Shewanella algae* with the accession number CP033574), *Vibrio alginolyticus* (Ye et al., 2016), *Pseudomonas aeruginosa* (Naas et al., 1999), *Achromobacter xylosoxidans* (with the accession number DQ393569), and *Pasteurella multocida* (with the accession number CP014157), but not in *W. chitiniclastica*. Generally, *bla*_VEB–1_ is carried by integrons, and *aadB* encoding an aminoglycoside adenyltransferase is located downstream of *bla*_VEB–1_ (Naas et al., 1999, 2000).

This study showed the complete genome sequences of a *W. chitiniclastica* from the pancreas of a dead zebra in China, which carried one chromosome and one novel type of *bla*_VEB–1_-carrying plasmid. Detailed genetic dissection was applied to this plasmid to display the genetic environment of *bla*_VEB–1_. The transcription start site and the promoter of the *bla*_VEB–1_ region were identified. The data presented here provided a deeper genomics and bioinformatics understanding of *W. chitiniclastica* for clinical treatment and pathogenesis research. To the best of our knowledge, this is the first report of the genetic characterization of a novel *bla*_VEB–1_-carrying plasmid from *W. chitiniclastica*.

## 2. Materials and methods

### 2.1. Bacterial isolation

The blood, heart, liver, spleen, lung, kidney, and pancreas specimens from a dead zebra were collected from Shenzhen Safari Park in 2013 and transferred to our laboratory in Changchun under sterile conditions for bacterial isolation. For each specimen, the tissue was plated onto brain heart infusion agar medium, 5% sheep blood agar medium, and MacConkey agar medium. The sample was then incubated at 37°C for 18 h under aerobic conditions.

The *W. chitiniclastica* strain DSM 18708 (Leibniz Institute DSMZ-German Collection of Microorganisms and Cell Cultures GmbH, Germany), isolated from artificial culture of *W. magnifica* in Hungary, was used as the reference strain in this study (Kõljalg et al., 2015).

### 2.2. Bacterial identification and phenotypic characteristics

Gram staining, BD Phoenix™-100 Automated Microbiology System (Becton, Dickinson and Company, USA) detection, PCR amplification of the 16S rRNA, *rpoB*, and *gyrB* genes followed by sequencing, and matrix-assisted laser desorption ionization-time of flight mass spectrometry (MALDI-TOF MS, Bruker Daltonics, USA) were conducted for bacterial identification.

A transmission electron microscopy investigation was carried out. The motility of the bacteria was tested on motility agar (Tittsler and Sandholzer, 1936). To determine the bacterial growth curves, the optical density at 600 nm (OD_600_) was monitored for each culture with a 1-h interval during the first 12 h and a 2-h interval during the last 12 h using the NanoPhotometer N60 (Implen, Germany).

The minimum inhibitory concentrations (MICs) of piperacillin-tazobactam, ceftazidime, cefotaxime, cefepime, aztreonam, imipenem, meropenem, amikacin, gentamicin, ciprofloxacin, levofloxacin, tetracycline, chloramphenicol, and trimethoprim-sulfamethoxazole were determined using the BD Phoenix™-100 Automated Microbiology System. The *E. coli* ATCC 25922 was used as an internal quality control.

The biochemical characteristics were tested using the BD Phoenix™-100 Automated Microbiology System, and the cellular fatty acid composition was detected using gas chromatography (Hewlett Packard 6890, USA) and identified using the Sherlock Microbial Identification System (MIDI, USA).

### 2.3. Sequencing and sequence assembly

The bacterial genomic DNA was isolated using the UltraClean microbial kit (Qiagen, Germany) and sequenced with a PacBio RS II sequencer (Pacific Biosciences, USA) (Fu et al., 2022; Li et al., 2022). The reads were assembled *de novo* using SMARTdenovo.^1^

### 2.4. Bacterial precise species identification

Bacterial precise species identification was conducted using pairwise average nucleotide identity (ANI) analysis between the bacterial genomic DNA sequenced in this study and the reference genome.^2^ A cutoff of ≥ 95% ANI was used to define a bacterial species (Richter and Rosselló-Móra, 2009).

### 2.5. Phylogenetic analysis

The core genes and specific genes of the bacterial genomes were analyzed using the CD-HIT rapid clustering of similar proteins software with a threshold of 50% pairwise identity and 0.7 length difference cutoff in amino acid (Li et al., 2001, 2002; Li and Godzik, 2006). The core amino acid sequences were extracted and aligned using MUSCLE (Edgar, 2004). Based on the core amino acid sequences, a maximum-likelihood phylogenetic tree was constructed using TreeBeST^3^ with a bootstrap iteration of 1,000.

### 2.6. Sequence annotation

RAST 2.0 (Brettin et al., 2015), blastp/blastn (Boratyn et al., 2013), and DANMEL (Wang et al., 2022) searches were used to predict open reading frames (ORFs). The online databases CARD (Jia et al., 2017), ResFinder (Zankari et al., 2012), ISfinder (Siguier et al., 2006), and INTEGRALL (Moura et al., 2009) were used to find resistance genes and mobile elements. Inkscape 1.0 was used to draw gene organization diagrams.^4^

### 2.7. Identification of the transcription start site and the promoter of the *bla*_VEB–1_ region

The total RNA of the bacterial isolates was extracted using the RNeasy maxi kit (Qiagen, Germany). Rapid amplification of cDNA ends (RACE) was performed using 5′-RACE system version 2.0 (Invitrogen, USA) according to the manufacturer’s instructions. Three gene-specific primers GSP1 (5′-ATCCTTCTCATTGCTG-3′), GSP2 (5′-CTCCTATTCTGGCATTTTTTG-3′), and GSP3 primer (5′-AAGTTGTCAGTTTGAGCATTT-3′) were used. The final PCR products were cloned into the pMD18-T vector, and then, the positive clones were identified and sequenced. The 5′-RACE experiment was repeated three times. Five clones were selected randomly and sequenced each time. The transcription start site was determined according to the sequence comparison between the positive clones and the *bla*_VEB–1_ region. The online database BPROM (Cassiano and Silva-Rocha, 2020) was used for promoter prediction.

### 2.8. Conjugation and electroporation experiments

Conjugal transfer and electroporation were performed as described previously (Liang et al., 2018; Guan et al., 2022).

### 2.9. Data availability statement

The complete sequences of the chromosome of BM-Y and the plasmid pBM-Y were submitted to GenBank under the accession numbers CP115969 and CP115970, respectively.

## 3. Results

### 3.1. Identification of *W. chitiniclastica* BM-Y and its phenotypic characteristics

BM-Y was isolated from the pancreas specimens of a dead zebra. According to Gram staining and the sequences of the amplicon of the 16S rRNA, *rpoB*, and *gyrB* genes, BM-Y was identified as the Gram-negative bacteria *W. chitiniclastica*. The MALDI-TOF MS score of BM-Y was 2.418, confirming *W. chitiniclastica*. Scores above 2.300 represent a highly probable species identification. No other bacterial isolates were discovered in this study. BM-Y has a 97.19% ANI value with the reference genome DSM 18708 (accession number AQXD01000000). BM-Y contained one chromosome (2.0 Mb in length) and one plasmid pBM-Y (42.3 Kb in length), and they were fully sequenced herein. The chromosome carried no resistance genes. The plasmid pBM-Y harbored *bla*_VEB–1_, *aadB*, *aacA1*, and *tetA*(H).

**FIGURE 1:**
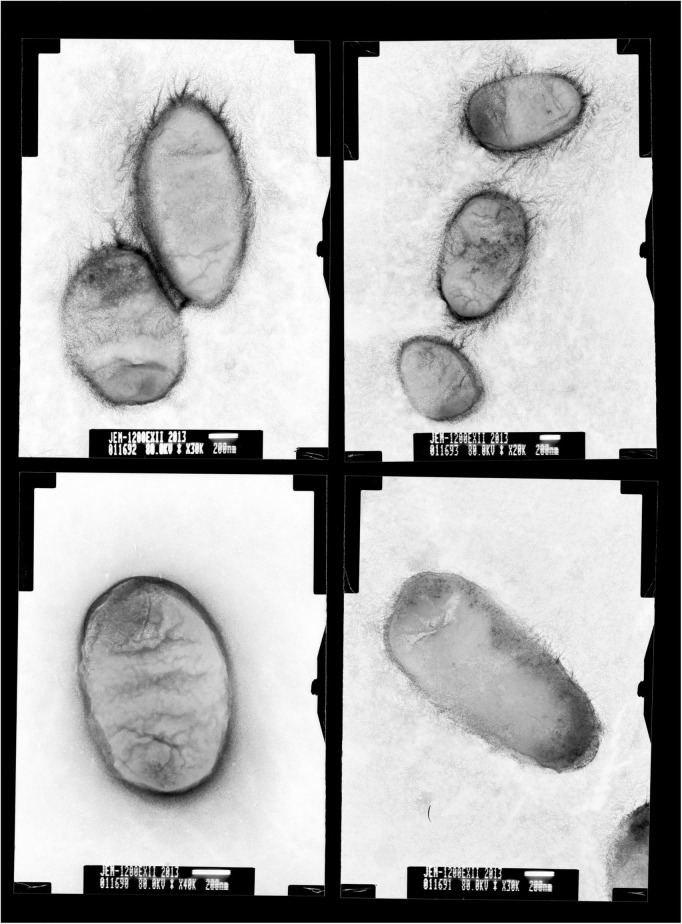
Transmission electron micrographs of *Wohlfahrtiimonas chitiniclastica* BM-Y. BM-Y was incubated on a brain heart infusion agar medium at 37°C for 18 h.

Colonies of BM-Y were small (colony diameter 0.8–1.0 mm), entire, convex, smooth, and glistening and were composed of short, straight, and non-motile rods (0.1–0.2 × 1.0–1.5 μm, Figure 1), which did not produce hemolysin on 5% sheep blood agar medium.

**FIGURE 2:**
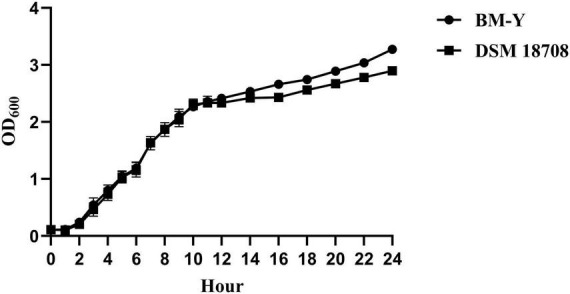
Bacterial growth curves of *W. chitiniclastica* BM-Y and DSM 18708.

Both BM-Y and DSM 18708 reached the middle logarithmic phase (OD_600_ value of about 1.0) and the stationary stage at the fifth hour and the 12th hour, respectively (Figure 2).

The MICs of ceftazidime, cefotaxime, cefepime, aztreonam, gentamicin, and tetracycline of BM-Y were higher than that of DSM 18708. The MICs of 14 antimicrobial drugs are shown in Table 1.

**TABLE 1:** Minimum inhibitory concentrations of 14 antimicrobial drugs.

Antibiotics	Minimum inhibitory concentration (MIC, μg/mL)		
	**BM-Y**	**DSM 18708**	**ATCC 25922**
Piperacillin-tazobactam	< 4/4	> 64/4	< 4/4
Ceftazidime	> 16	16	< 1
Cefotaxime	8	< 1	< 1
Cefepime	> 16	8	< 2
Aztreonam	> 16	8	< 2
Imipenem	< 1	< 1	< 1
Meropenem	< 1	< 1	< 1
Amikacin	16	16	< 8
Gentamicin	8	4	< 2
Ciprofloxacin	< 0.5	< 0.5	< 0.5
Levofloxacin	< 1	< 1	< 1
Tetracycline	> 8	< 2	< 2
Chloramphenicol	< 4	< 4	> 16
Trimethoprim/ sulfamethoxazole	< 0.5/9.5	< 0.5/9.5	< 0.5/9.5

The biochemical test results of BM-Y and DSM 18708 are shown in Table 2. A varied reaction between these two strains was observed for acetate, polymyxin B, and glycine. The cellular fatty acid profile (Table 3) of BM-Y revealed a proportionally high level of C_18:1_ω7*c*, C_14:0_, and C_16:0_, which was consistent with DSM 18708.

**TABLE 2:** Biochemical characteristics of BM-Y and DSM 18708.

Substrates	BM-Y	DSM 18708
Arginine	−	−
Glutaryl-glycine-arginine	−	−
L-leucine	+	+
L-pyroglutamic acid	−	−
Acetate	−	+
Colistin	+	+
Malonate	−	−
4MU-N-acetyl-BD-glucosaminide	−	−
PNP-BD-glucoside	−	−
β-gentiobiose	−	−
D-galactose	−	−
Sorbitol	−	−
L-arabinose	−	−
Maltulose	−	−
Ornithine	−	−
Glycine-proline	−	−
L-arginine	−	−
L-phenylalanine	+	+
L-tryptophan	−	−
Adonitol	−	−
D-mannitol	−	−
Polymyxin B	−	+
γ-glutamyl-NA	−	−
Bis (PNP) phosphate	+	+
Dextrose	−	−
D-gluconic acid	−	−
Sucrose	−	−
L-rhamnose	−	−
N-acetyl-galactosamine	−	−
Urea	−	−
Glycine	−	+
L-glutamic acid	−	−
L-proline	−	−
Lysine-alanine	−	−
Citrate	−	−
α-ketoglutaric acid	+	+
Tiglic acid	−	−
L-proline-NA	+	+
β-allose	−	−
D-fructose	−	−
D-melibiose	−	−
Galacturonic acid	−	−
Methyl-B-glucoside	−	−
N-acetyl-glucosamine	−	−
Esculin	−	−

+, positive; −, negative.

**TABLE 3:** Cellular fatty acid profiles of BM-Y and DSM 18708.

Fatty acids		BM-Y (%)	DSM 18708 (%)
Saturated acids	C_12:0_	5.68	5.10
	C_14:0_	17.70	14.40
	C_16:0_	6.57	15.60
	C_18:0_	tr	1.30
Unsaturated	C18:1ω7*c*	50.03	47.30
Branched acids	C_12:0_ 3-OH	3.23	1.00
	cyclo-C_19:0_ω8*c*	5.68	7.60
Summed features	Summed feature 1	3.08	1.33
	Summed feature 2	5.64	2.86

tr, trace amount (< 0.5%). Summed features represent two or three fatty acids that could not be separated by the Microbial Identification System. Summed feature 1 comprises C_14:0_ 3-OH/iso-C_16:1_. Summed feature 2 comprises C_16:1_ω7c/iso-C_15_ 2-OH.

### 3.2. Phylogenetic analysis

**FIGURE 3:**
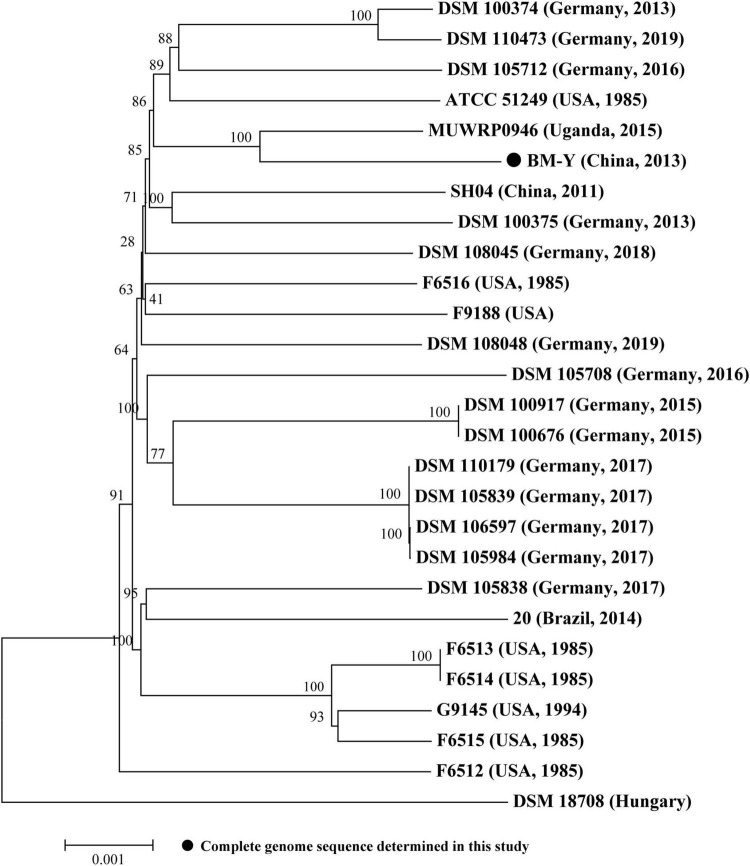
Maximum-likelihood phylogenetic tree of 27 *W. chitiniclastica* isolates. The numbers above branches indicate bootstrap values of 1,000 times. The bar corresponds to a scale of sequence divergence.

Based on the bacterial core/pan-genome analysis, we conducted the phylogenetic analysis of 27 sequenced *W. chitiniclastica* isolates (Supplementary Table 1), including one complete genome sequence from this study and 26 draft genome sequences from GenBank (last accessed March 17, 2023). Construction of a phylogenetic tree (Figure 3) based on core genes (1602/3597, 44.53%) of 27 *W. chitiniclastica* genomes revealed that all strains clustered in one subclade with the reference genome DSM 18708, and BM-Y shared the closest genetic relationship with MUWRP0946 from Uganda. This result indicated a surprisingly conserved core/pan genome without clear host or geographical clustering, suggesting a potential spread and transmission. This is in line with Kopf’s report (Kopf et al., 2022).

### 3.3. Organization of the *bla*_VEB–1_-carrying plasmid pBM-Y

**FIGURE 4:**
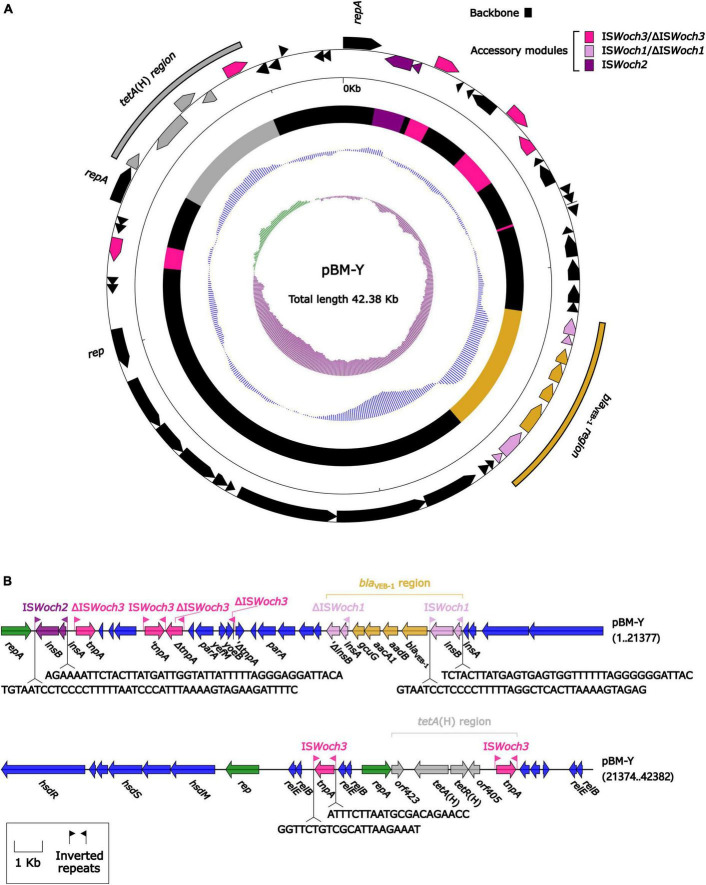
Organization of the plasmid pBM-Y. **(A)** Schematic map of pBM-Y. Genes are denoted by arrows, and the backbone and accessory module regions are highlighted in black and color, respectively. The innermost circle presents the GC-skew [(G–C)/(G+C)], with a window size of 500 bp and a step size of 20 bp. The next-to-innermost circle presents the GC content. **(B)** Linear structure of pBM-Y. Genes are denoted by arrows. The genes, accessory genetic elements (AGEs), and other features are colored based on their functional classification. The numbers in brackets indicate nucleotide positions within pBM-Y.

The modular structure of pBM-Y (Figure 4) was separated into the backbone and the accessory modules. The backbone contained three different *rep* (replication) with no similar DNA sequences (nucleotide identity < 95%) in GenBank, two *parA* (partition) with different sequences, three groups of *relBE* (a toxin–antitoxin system) with low nucleotide identities, one group of *yefM*/*yoeB* (a toxin–antitoxin system), and one group of *hsdMSR* (a type I restriction–modification system). No conjugal transfer genes were identified in the backbone. The accessory modules were composed of a *bla*_VEB–1_ region, a *tetA*(H) region, and intact or truncated insertion sequences (ISs) IS*Woch2* and IS*Woch3*.

**FIGURE 5:**
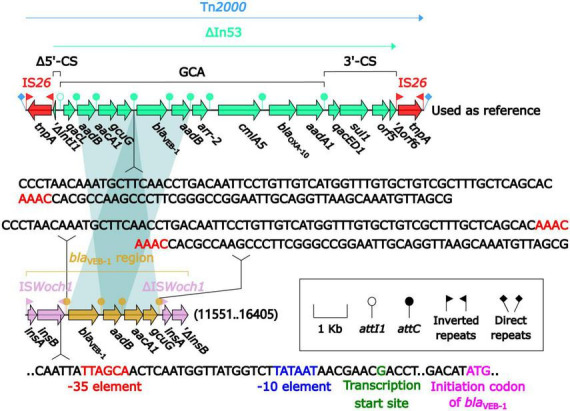
Comparison of the *bla*_VEB–1_ region from pBM-Y and *bla*_VEB–1_-carrying Tn*2000*. Genes are denoted by arrows. The genes, AGEs, and other features are colored based on their functional classification. The shading in light blue denotes regions of homology (nucleotide identity ≥ 95%). The 3′ fragment of *attC*_*aacA1*/*gcuG* is upstream of *bla*_VEB–1_, while the 5′ fragment is downstream of *gcuG*. The numbers in the brackets indicate the nucleotide positions within pBM-Y. Tn*2000* (Naas et al., 2001) (accession number AF205943) was used as a reference.

*bla*_VEB–1_ was originally found in Tn*2000* in the plasmid pNLT1 from *E. coli* MG-1 (Poirel et al., 1999; Naas et al., 2001). Tn*2000* was an IS*26*-composite transposon composed of ΔIn53 and was used as a reference herein. A detailed sequence comparison (Figure 5) was applied to the *bla*_VEB–1_-carrying ΔIn53 from Tn*2000* and the *bla*_VEB–1_ region from pBM-Y. Both ΔIn53 and the *bla*_VEB–1_ region harbored the *bla*_VEB–1_ cassette (with a 133-bp attachment site of the cassette, *attC*), the *aadB* cassette (with a 60-bp *attC*), and the *aacA1*/*gcuG* fusion gene cassette (with a 118-bp *attC*) (Naas et al., 2001), but they showed three major modular variations: (i) an intact *attC*_*aacA1*/*gcuG* in ΔIn53, while an interrupted one in the *bla*_VEB–1_ region; (ii) a gene cassette array (GCA) *qacL*–*aadB*–*aacA1*–*gcuG*–*bla*_VEB–1_–*aadB*–*arr-2*–*cmlA5*–*bla*_OXA–10_–*aadA1* in ΔIn53, while a GCA *bla*_VEB–1_–*aadB*–*aacA1*–*gcuG* in the *bla*_VEB–1_ region; and (iii) a truncated 5′-conserved segment (5′-CS) and a 3′-CS were upstream and downstream, respectively, of the GCA in ΔIn53, while a novel IS element IS*Woch1* and a truncated one were upstream and downstream, respectively, of the GCA in the *bla*_VEB–1_ region. No direct repeats (DRs) were identified at the ends of the *bla*_VEB–1_ region from pBM-Y.

**FIGURE 6:**
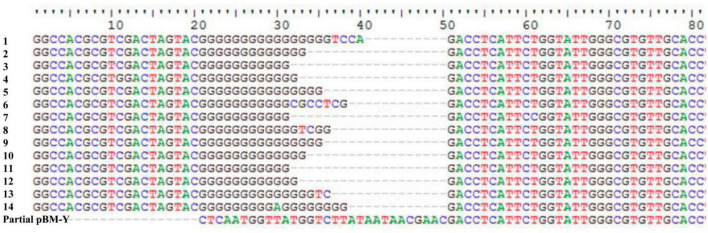
Identification of the transcription start site of the *bla*_VEB–1_ region. Multiple sequence alignments of 14 positive clones and partial pBM-Y.

IS*Woch1*, IS*Woch2*, and IS*Woch3* were named based on the first four letters of the species in which they were first discovered (*Wohlfahrtiimonas chitiniclastica*) and had IR of 37, 45, and 21 bp, respectively (Figure 4). IS*Woch1* and IS*Woch2* encoded two transposases, while IS*Woch3* carried a single one. No DNA sequences similar (nucleotide identity < 95%) to these transposases were found using blastn analysis. The promoter of IS*Woch1* was determined using 5′-RACE experiments. Fourteen of 15 positive clones were successfully sequenced. GACCT (Figure 6), the transcription start site of the *bla*_VEB–1_ region, was identified through multiple sequence alignments. This site was found in IS*Woch1* and located 173 bp upstream of the initiation codon of *bla*_VEB–1_. A promoter with the −10/−35 region (TATAAT/TTAGCA) was located upstream of this site (Figure 5). The spacing between the −10/−35 region was 18 bp. Based on the results of the promoter prediction, IS*Woch2* contained a −10/−35 region (TATCAT/ATACCA, with a 21-bp spacer), and IS*Woch3* carried a −10/−35 region (TAAAAT/TTATCA, with a 19-bp spacer). IS*Woch1*–*bla*_VEB–1_–*aadB*–*aacA1*–*gcuG*–ΔIS*Woch1* was composed of the *bla*_VEB–1_ region (Figure 5). IS*Woch2* was located upstream of the truncated IS*Woch3* and downstream of the *repA*. Three intact IS*Woch3* and three truncated ones were found in pBM-Y. IS*Woch3*–*orf405*–*tetR*(H)–*tetA*(H)–*orf423* formed the *tetA*(H) region.

### 3.4. Conjugation and electroporation experiments

The *bla*_VEB–1_-carrying transconjugants could not be obtained, regardless of the number of times the conjugation experiments were performed. This result might be due to the absence of the essential conjugal transfer genes, including *rlx* (relaxase), *oriT* (origin of conjugative replication), *pri* (DNA primase), and *cpl* (coupling protein), and the type IV secretion system (T4SS) in pBM-Y.

The *bla*_VEB–1_-carrying electroporants were not obtained, no matter how many times the electroporation experiments were conducted. Because we failed to extract the plasmid pBM-Y from strain BM-Y.

## 4. Discussion

*Wohlfahrtiimonas chitiniclastica* is an emerging fly-borne zoonotic pathogen, which is often carried by different species of flies, and it causes infections in immunocompromised patients and some animals (Almuzara et al., 2011; Thaiwong et al., 2014), leading to bacteremia, sepsis, and other infections. β-lactams, quinolones, and trimethoprim/sulfamethoxazole are used to treat patients and animals infected by *W. chitiniclastica* (Schröttner et al., 2017).

Herein, BM-Y was isolated from the pancreas of a dead zebra in Shenzhen Safari Park in China in 2013. We speculated that *W. chitiniclastica* was possibly transferred to this park through either or both internal and international transport routes. Notably, *W. chitiniclastica* was first isolated and identified in China from *C. megacephala* captured from the Pudong International Airport in 2011 (Cao et al., 2013). This fly is one of the most common species found in South China (Liu et al., 2009), and it may have become the depositor of *W. chitiniclastica* in China. However, BM-Y shares the closest genetic relationship with MUWRP0946 from Uganda. *W. chitiniclastica* is more likely to be transferred from other countries to this park through international food (animal- and plant-based) trade or travel. We failed to isolate *W. chitiniclastica* from the flies collected in Shenzhen Safari Park, although we made several attempts.

We are not sure whether BM-Y was associated with the death of the zebra because we failed to find the maggots from the zebra. Generally, flies transmit *W. chitiniclastica* to the host by laying eggs that subsequently hatch into larvae inside an open wound (Schröttner et al., 2017). In this study, neither an open wound nor a maggot was found, but the flies might have carried *W. chitiniclastica* to deposit the eggs on the mucosal surfaces of the zebra (Schröttner et al., 2017). It is necessary to continuously monitor the spread of *W. chitiniclastica*, especially the ones that carry antimicrobial resistance genes, in the future.

pBM-Y is a very specific plasmid. It carries three different and novel *rep* genes. Currently, no other similar genes can be found in GenBank. pBM-Y encodes two toxin–antitoxin systems: RelBE and YefM/YoeB, which have been shown to increase plasmid maintenance (Gotfredsen and Gerdes, 1998). The HsdMSR type I restriction–modification system is also identified in pBM-Y. This system can stabilize plasmids by degrading the unmethylated incoming DNA (Oliveira et al., 2014). It is speculated herein that the loss of the toxin–antitoxin or restriction–modification system might lead to the instability of pBM-Y. No conjugative genes were identified; so, pBM-Y is putatively mobilized by the conjugative plasmids (Xu et al., 2021).

The MICs of ceftazidime, cefotaxime, cefepime, aztreonam, gentamicin, and tetracycline of BM-Y were higher than that of DSM 18708, which was related to the resistance genes *bla*_VEB–1_, *aadB*, *aacA1*, and *tetA*(H). These genes were identified in pBM-Y using the CARD and ResFinder online databases. This indicates that these genes can express effectively and are involved in the increase in MICs. The *bla*_VEB–1_ gene cassette, the *aadB* gene cassette, and the *aacA1*/*gcuG* fusion cassette are individual mobile units and are usually found inserted into an integron (Partridge et al., 2018). In this study, they formed a GCA (*bla*_VEB–1_–*aadB*–*aacA1*–*gcuG*) different from that (*qacL*–*aadB*–*aacA1*–*gcuG*–*bla*_VEB–1_–*aadB*–*arr-2*–*cmlA5*–*bla*_OXA–10_–*aadA1*) in ΔIn53 from Tn*2000* (Naas et al., 2001). This demonstrates that these gene cassettes might undergo reassembly via homologous recombination. An intact and a truncated IS*Woch1* were at the 5′ and 3′ ends of *bla*_VEB–1_–*aadB*–*aacA1*–*gcuG* in pBM-Y, respectively, which indicated that this GCA might be captured by IS*Woch1* to form a translocatable unit (TU, one copy of IS*Woch1* and an adjacent region) (Partridge et al., 2018). Then, this TU inserts into a recipient that lacks IS*Woch1* (intermolecular replicative transposition) or targets an existing copy of IS*Woch1* (intermolecular conservative transposition) (Partridge et al., 2018). However, one copy of IS*Woch1* has been truncated during transposition.

Two copies of IS*Woch1* participated in the movement of *bla*_VEB–1_–*aadB*–*aacA1*–*gcuG*. A single IS can also move an adjacent region that includes one or more resistance genes by forming a TU (Partridge et al., 2018). Herein, IS*Woch3* was located upstream of *orf405*–*tetR*(H)–*tetA*(H)–*orf423*, indicating that it might be involved in the mobilization of *tetA*(H), and this *tetA*(H) region might preferentially insert next to an existing copy of IS*Woch3* in the recipient molecule, generating a structure of IS*Woch3*–*orf405*–*tetR*(H)–*tetA*(H)–*orf423*–IS*Woch3* (Partridge et al., 2018). It should also be noted that six intact or truncated copies of IS*Woch3* were presented in pBM-Y, demonstrating that they participated in complex homologous recombination events and promoted the assembly of complex structures as observed in pBM-Y. A promoter was found or predicted in each of IS*Woch1*, IS*Woch2*, and IS*Woch3*, meaning these three ISs might provide a promoter for captured genes. Taken together, IS*Woch1*, IS*Woch2*, and IS*Woch3* might promote the dissemination of drug resistance genes and affect the expression of these genes to regulate antimicrobial resistance (Noel et al., 2022). Surveillance studies for these three ISs are necessary.

The promoter region (containing the −10 and −35 elements) of the *bla*_VEB–1_ region was located in IS*Woch1*. The optimal spacing between the −10 and 35 elements is 16–18 bp (Aoyama et al., 1983), and in this study, it was 18 bp. This indicates that IS*Woch1* provides a strong promoter for the *bla*_VEB–1_ region. Therefore, BM-Y can express β-lactamase and show resistance to ceftazidime, cefepime, and aztreonam. This means that some β-lactams are not suitable for treating infections caused by *bla*_VEB–1_-carrying *W. chitiniclastica*, while quinolones and trimethoprim/sulfamethoxazole can still be the first choices.

To the best of our knowledge, this is the first report of the genetic characterization of a novel *bla*_VEB–1_-carrying plasmid with three new ISs from *W. chitiniclastica*. The acquisition of this plasmid can give bacteria the fitness advantage for adapting to mammals and enable bacteria to acquire new antimicrobial resistance genes. The resistance of *W. chitiniclastica* to ceftazidime, cefepime, aztreonam, and tetracycline might enhance its ability to survive under drug selection pressure and aggravate the difficulty in treating infections caused by *W. chitiniclastica*. It is necessary to continuously monitor the spread of the *bla*_VEB–1_-carrying *W. chitiniclastica* and the possibility of acquiring other drug resistance genes in *W. chitiniclastica*.

## Data availability statement

The datasets presented in this study can be found in online repositories. The names of the repository/repositories and accession number(s) can be found in this article/Supplementary material.

## Ethics statement

The animal study was approved by the Laboratory Animal Welfare and Ethics Committee of the Changchun Veterinary Research Institute, Chinese Academy of Agricultural Sciences. The study was conducted in accordance with the local legislation and institutional requirements.

## Author contributions

JiaG: Formal analysis, Investigation, Writing – original draft. WZ: Formal analysis, Writing – original draft. JinG: Formal analysis, Writing – original draft. LZhe: Investigation, Writing – original draft. GL: Formal analysis, Writing – original draft. FH: Resources, Writing – original draft. ML: Formal analysis, Writing – original draft. XJ: Investigation, Writing – original draft. YS: Investigation, Writing – original draft. LZhu: Conceptualization, Writing – review and editing. XG: Conceptualization, Writing – review and editing.

## Footnotes

1
http://github.com/ruanjue/smartdenovo

2
http://www.ezbiocloud.net/tools/ani

3
http://treesoft.sourceforge.net/treebest.shtml

4
http://inkscape.org/en/
