# Supplementary material for: A Wohlfahrtiimonas chitiniclastica with a novel type of blaVEB–1-carrying plasmid isolated from a zebra in China
Source: Front Microbiol. 2023 Nov 2;14:1276314. doi: 10.3389/fmicb.2023.1276314 (PMC10656743; doi:10.3389/fmicb.2023.1276314)
Supplement: Supplementary file 1 [file Table_1.DOCX]

Supplementary Material

# Supplementary Table

**TABLE S1** Background information of 27 *Wohlfahrtiimonas* *chitiniclastica* isolates.

| **Isolate** | **Bacterium** | **Country** | **Year** | **Specimen** | **Host** | **Source** | **Complete/draft genome sequence** | **Accession number** | | **Reference** |
| --- | --- | --- | --- | --- | --- | --- | --- | --- | --- | --- |
| BM-Y | *W. chitiniclastica* | China | 2013 | Pancreas | Zebra | This study | Complete | Chromosome | CP115969 | This study |
|  |  |  |  |  |  |  |  | pBM-Y | CP115970 |  |
| SH04 | *W. chitiniclastica* | China | 2011 | Fly | Fly | GenBank | Draft | AOBV01000000 | | (Cao et al., 2013) |
| ATCC 51249 | *W. chitiniclastica* | USA | 1985 | Arm | Homo sapiens | GenBank | Draft | NEFL01000000 | |  |
| F6512 | *W. chitiniclastica* | USA | 1985 | Foot | Homo sapiens | GenBank | Draft | NEFK01000000 | |  |
| F6513 | *W. chitiniclastica* | USA | 1985 | Leg | Homo sapiens | GenBank | Draft | NEFJ01000000 | |  |
| F6514 | *W. chitiniclastica* | USA | 1985 | Oral lesion | Homo sapiens | GenBank | Draft | NEFI01000000 | |  |
| F6515 | *W. chitiniclastica* | USA | 1985 | Ankle | Homo sapiens | GenBank | Draft | NEFH01000000 | |  |
| F6516 | *W. chitiniclastica* | USA | 1985 | Foot | Homo sapiens | GenBank | Draft | NEFG01000000 | |  |
| F9188 | *W. chitiniclastica* | USA |  | Leg wound | Homo sapiens | GenBank | Draft | NEFE01000000 | |  |
| G9145 | *W. chitiniclastica* | USA | 1994 | Wound | Homo sapiens | GenBank | Draft | NEFC01000000 | |  |
| DSM 18708 | *W. chitiniclastica* | Hungary |  |  | Fly | GenBank | Draft | AQXD01000000 | | (Tóth et al., 2008) |
| DSM 100374 | *W. chitiniclastica* | Germany | 2013 |  | Homo sapiens | GenBank | Draft | JAGIBR010000000 | | (Schröttner et al., 2017) |
| DSM 100375 | *W. chitiniclastica* | Germany | 2013 |  | Homo sapiens | GenBank | Draft | JAGIBS010000000 | | (Schröttner et al., 2017) |
| DSM 100676 | *W. chitiniclastica* | Germany | 2015 |  | Homo sapiens | GenBank | Draft | JAGIBT010000000 | | (Schröttner et al., 2017) |
| DSM 100917 | *W. chitiniclastica* | Germany | 2015 |  | Homo sapiens | GenBank | Draft | JAGIBU010000000 | | (Schröttner et al., 2017) |
| DSM 105708 | *W. chitiniclastica* | Germany | 2016 |  | Homo sapiens | GenBank | Draft | JAGIBV010000000 | | (Kopf et al., 2021) |
| DSM 105712 | *W. chitiniclastica* | Germany | 2016 |  | Homo sapiens | GenBank | Draft | JAGIBW010000000 | | (Kopf et al., 2021) |
| DSM 105838 | *W. chitiniclastica* | Germany | 2017 |  | Homo sapiens | GenBank | Draft | JAGIBX010000000 | | (Kopf et al., 2021) |
| DSM 105839 | *W. chitiniclastica* | Germany | 2017 |  | Homo sapiens | GenBank | Draft | JAGIBY010000000 | | (Kopf et al., 2021) |
| DSM 105984 | *W. chitiniclastica* | Germany | 2017 |  | Homo sapiens | GenBank | Draft | JAGIBZ010000000 | | (Kopf et al., 2021) |
| DSM 106597 | *W. chitiniclastica* | Germany | 2017 |  | Homo sapiens | GenBank | Draft | JAGICA010000000 | | (Kopf et al., 2021) |
| DSM 108045 | *W. chitiniclastica* | Germany | 2018 |  | Homo sapiens | GenBank | Draft | JAGICB010000000 | | (Kopf et al., 2021) |
| DSM 108048 | *W. chitiniclastica* | Germany | 2019 |  | Homo sapiens | GenBank | Draft | JAGICC010000000 | | (Kopf et al., 2021) |
| DSM 110179 | *W. chitiniclastica* | Germany | 2017 |  | Homo sapiens | GenBank | Draft | JAGICD010000000 | | (Kopf et al., 2021) |
| DSM 110473 | *W. chitiniclastica* | Germany | 2019 |  | Homo sapiens | GenBank | Draft | JAGICE010000000 | | (Kopf et al., 2021) |
| 20 | *W. chitiniclastica* | Brazil | 2014 | Chicken carcass |  | GenBank | Draft | LWST01000000 | | (Matos et al., 2016) |
| MUWRP0946 | *W. chitiniclastica* | Uganda | 2015 | Wound | Homo sapiens | GenBank | Draft | JANDKC020000000 | | (Byarugaba et al., 2023) |

**References**

Byarugaba, D.K., Erima, B., Wokorach, G., Najjuka, F., Kiyengo, J., Kwak, Y.I., et al. (2023). Genome sequence analysis of a *Wohlfahrtiimonas* *chitiniclastica* strain isolated from a septic wound of a hospitalized patient in Uganda. *Microbiology Resource Announcements*. e0084022. doi: 10.1128/mra.00840-22.

Cao, X., Chen, T., Xu, L., Yao, L., Qi, J., Zhang, X., et al. (2013). Complete genome sequence of *Wohlfahrtiimonas* *chitiniclastica* strain SH04, isolated from *Chrysomya* *megacephala* collected from Pudong International Airport in China. *Genome Announcements*. 1(2), e0011913. doi: 10.1128/genomeA.00119-13.

Kopf, A., Bunk, B., Coldewey, S.M., Gunzer, F., Riedel, T., and Schröttner, P. (2021). Identification and antibiotic profiling of *Wohlfahrtiimonas* *chitiniclastica*, an underestimated human pathogen. *Frontiers In Microbiology*. 12, 712775. doi: 10.3389/fmicb.2021.712775.

Matos, J., Queiroga, A.P., de Oliveira Pedroza Bindi dos Reis, C.C., Ribeiro, R.L., Teixeira, L.M., Albano, R.M., et al. (2016). First report of the emerging zoonotic agent *Wohlfahrtiimonas* *chitiniclastica* isolated from a retail frozen chicken in Rio de Janeiro, Brazil. *Antonie Van Leeuwenhoek*. 109(5), 729-734. doi: 10.1007/s10482-016-0673-x.

Schröttner, P., Rudolph, W.W., Damme, U., Lotz, C., Jacobs, E., and Gunzer, F. (2017). *Wohlfahrtiimonas* *chitiniclastica*: current insights into an emerging human pathogen. *Epidemiology and Infection*. 145(7), 1292-1303. doi: 10.1017/S0950268816003411.

Tóth, E.M., Schumann, P., Borsodi, A.K., Kéki, Z., Kovács, A.L., and Márialigeti, K. (2008). *Wohlfahrtiimonas* *chitiniclastica* gen. nov., sp. nov., a new gammaproteobacterium isolated from *Wohlfahrtia* *magnifica* (Diptera: Sarcophagidae). *International Journal of Systematic and Evolutionary Microbiology*. 58(Pt 4), 976-981. doi: 10.1099/ijs.0.65324-0.
